# Bone mass of female dance students prior to professional dance training: A cross-sectional study

**DOI:** 10.1371/journal.pone.0180639

**Published:** 2017-07-05

**Authors:** Tânia Amorim, George S. Metsios, Matthew Wyon, Alan M. Nevill, Andreas D. Flouris, José Maia, Eduardo Teixeira, José Carlos Machado, Franklim Marques, Yiannis Koutedakis

**Affiliations:** 1 Centre of Research, Education, Innovation and Intervention in Sport, Faculty of Sports, University of Porto, Porto, Portugal; 2 The Faculty of Education, Health and Wellbeing, University of Wolverhampton, Walsall, United Kingdom; 3 School of Exercise Sciences, University of Thessaly, Trikala, Greece; 4 National Institute of Dance Medicine and Science, London, United Kingdom; 5 FAME Laboratory, Department of Exercise Science, University of Thessaly, Trikala, Greece; 6 Research Center in Physical Activity, Health and Leisure, Faculty of Sports, University of Porto, Porto, Portugal; 7 Institute of Molecular Pathology and Immunology of the University of Porto, Porto, Portugal; 8 Faculty of Pharmacy, University of Porto, Porto, Portugal; 9 Institute for Research and Technology–Thessaly, CERTH, Trikala, Greece; Georgia Regents University, UNITED STATES

## Abstract

**Background:**

Professional dancers are at risk of developing low bone mineral density (BMD). However, whether low BMD phenotypes already exist in pre-vocational dance students is relatively unknown.

**Aim:**

To cross-sectionally assess bone mass parameters in female dance students selected for professional dance training (first year vocational dance students) in relation to aged- and sex-matched controls.

**Methods:**

34 female selected for professional dance training (10.9yrs ±0.7) and 30 controls (11.1yrs ±0.5) were examined. Anthropometry, pubertal development (Tanner) and dietary data (3-day food diary) were recorded. BMD and bone mineral content (BMC) at forearm, femur neck (FN) and lumbar spine (LS) were assessed using Dual-Energy X-Ray Absorptiometry. Volumetric densities were estimated by calculating bone mineral apparent density (BMAD).

**Results:**

Dancers were mainly at Tanner pubertal stage I (vs. stage IV in controls, *p*<0.001), and demonstrated significantly lower body weight (*p*<0.001) and height (*p*<0.01) than controls. Calorie intake was not different between groups, but calcium intake was significantly greater in dancers (*p*<0.05). Dancers revealed a significantly lower BMC and BMD values at all anatomical sites (*p*<0.001), and significantly lower BMAD values at the LS and FN (*p*<0.001). When adjusted for covariates (body weight, height, pubertal development and calcium intake), dance students continued to display a significantly lower BMD and BMAD at the FN (*p*<0.05; *p*<0.001) at the forearm (*p*<0.01).

**Conclusion:**

Before undergoing professional dance training, first year vocational dance students demonstrated inferior bone mass compared to controls. Longitudinal models are required to assess how bone health-status changes with time throughout professional training.

## Introduction

Low bone mineral density (BMD) and osteoporosis are of major public health concern [1]. These conditions are characterised by low BMD and low bone mineral content (BMC), which lead to a fragile skeleton and increased risk of osteoporotic fractures [1]. Physical exercise is a key factor against the development of these conditions [2, 3], particularly weight-bearing exercises during the developmental years [4].

Although it is generally accepted that a moderate active lifestyle improves bone health, the effects of elite physical performance on bone health are not entirely clear [5, 6, 7]. Elite sports may have inherent several specific characteristics that might induce either beneficial or deleterious impact on bone metabolism [8]. In aesthetic sports, where leanness and control of body weight are essential requirements, the training loads that typically promote bone formation may be annulled [9–11]. For instance, concerns have been voiced over the possible negative effects of elite dance training demands on bone metabolism [12–14]. Indeed, female elite professional dancers are exposed to high levels of artistic and fitness strains, whilst aesthetic build and low body weight are embraced in dance culture [15–17]. In accordance, the majority of relevant studies report that professional female dancers have low BMD compared to controls or normative values [18], which increases the risk of developing osteoporosis in later life.

Low BMD and osteoporosis in adulthood may have paediatric antecedents, as bone mass during growth is the foundation for the adult skeleton [19]. Nevertheless, whether BMD differences between dancers and non-dancers do exist prior to professional dance training has to be confirmed. The aim of the present study was to assess levels of areal and volumetric measures of bone mass in first year female vocational dance students (selected for professional training).

## Methods

### Participants and study design

First year female students accepted in a vocational dance school (school that offers full-time dance training to become professional dancers; students have to audition for a place) during the academic years 2012/2013 and 2013/2014 were invited to participate in the study with no preliminary exclusion criteria; a total of 34 out of 48 (70.8%) volunteered. Control participants were recruited from a local state school by excluding those who participated or had previously participated in organised extracurricular physical activities; the total of 111 (28%) volunteered out of 391 eligible pupils. Of these 111 students, 30 were female and had the same chronological age as our dance students, and, therefore, were enrolled in the study. Amenorrheic/ oligomenorrheic pupils were not excluded. None of the participants (both dancers and controls) had received or were receiving medications/ supplementation known to affect bone metabolism. The mean age of dance students and controls was 10.9±0.7 yrs and 11.1±0.5 yrs, respectively. Participants and their guardians signed informed consents after reading a written explanation of the study and discussion with the investigators. The study was approved by the ethics committee of the Regional Administration of Health of Lisbon (Proc.063/CES/INV/2012).

Female dance students started their professional training in September (25 students in 2012 and 9 in 2013) and were assessed in December 2012 and 2013, respectively. According to published reports, a period of 3 months of physical exercise is not sufficient to induce bone mass gains in paediatric populations [20–22]. All participants were involved in 2 hours of physical education exercise twice a week; 80.1% of the included dance students had also taken recreational dance lessons on a weekly basis (1.8±0.7 hours per week). Specifically, 15.9% have been dancing for a year before vocational training, 23.8% for two years, 31.7% for four years and 28.6% for five years (they started these classes at the age of nine, eight, six and five, respectively). These dance classes were at a non-vocational level. At vocational level, dancers usually have 2–4 classes a day, and the training starts at the age of 10 and last for 8 years. The entrance to vocational level takes place through audition, which focuses on postural alignment, body characteristics, musically, coordination, as well as artistry. All participants described themselves as white Caucasian. Within the population of 34 dance students available for assessment, all underwent anthropometric measures, participated in bone measurements and reported Tanner stage, age at menarche, and menstrual history; only 32 (94.2%) completed a dietary questionnaire. Similarly, all 30 controls underwent anthropometric measures, participated in bone measurements and reported Tanner stage, age at menarche, and menstrual history, while 29 (96.7%) completed a dietary questionnaire.

### Anthropometry, maturation assessment, menstrual, energy expenditure, and nutritional analysis

Chronological age was obtained as decimal age (date of birth minus measurement date). Height and body mass were measured in t-shirt, shorts and bare feet using a stadiometer (Seca, Seca217 portable stadiometer, Hamburg, Germany) with accuracy of 0.1 cm and an electric scale (TANITA BC-418 MA Segmental Body Composition Analyser; Tanita Corporation, Tokyo, Japan) with an accuracy of 0.1 kg. All measurements were administered by the same investigator twice (mean of the two measurements were recorded); if the difference between the two measurements was greater than 0.3, a third measure was obtained. Pubertal development was assessed using Tanner staging (breast and public hair stage) by self-reporting. Standard line drawings and written descriptions were provided, and participants selected the picture that most accurately reflected their appearance [23]. All participants were presented with a questionnaire to determine age at menarche and regularity of menstrual cycles. Amenorrhea was defined as the absence of menses for three consecutive months, whereas oligomenorrhea was considered when menstrual cycles occurred at intervals of greater than 35 days. Nutrient intakes were recorded via a validated 3-day food diary [24]. Participants were asked to record all food and beverages consumed during two school days and one weekend day following appropriate instructions. The Food Processor SQL Edition, version 9.8.1 was used to estimate average energy and calcium intakes.

### Bone status measurements

BMD (g/cm^2^) and (BMC) (g) were determined for non-dominant forearm (33% radius), lumbar spine (L1-L4) (LS) and femoral neck (FN). To estimate volumetric density, bone mineral apparent density (BMAD) (g/cm^3^) was calculated for all sites using previously described formulas [25]. Vocational dance students and controls were assessed in two different centres using Dual-energy X-ray absorptiometry (DXA). DXA scans of dance students were performed using a GE Lunar Prodigy whereas DXA scans of controls were performed using a Hologic (Discovery Wi). The same certified technician conducted all scans and analyses at both centres. Although previous studies have demonstrated a high correlation between Lunar and Hologic DXA BMD measurements [26–28], there is a tendency for Lunar model to inflate BMD values by 15% compared to Hologic [29, 30, 31]. Therefore, in addition to the daily calibration required from each DXA manufacturer, cross-calibration of the two scanners was conducted using a group of 20 independent participants. These participants were measured with both Lunar and Hologic within a period of 5 days. Regression equations using BMD from Lunar as dependent variable and BMD from Hologic as independent variable were performed from the data obtained from the participants included from cross-calibration. Table 1 shows the correlation between the two DXA models (correlation was high).

**Table 1:** Correlation between the two DXA models.

	R	R^2^	Standard error of estimate
BMD measures			
Forearm	0.96	0.93	0.03
FN	0.97	0.93	0.05
LS	0.96	0.92	0.05
BMC measures			
Forearm	0.98	0.96	0.9
FN	0.94	0.88	0.46
LS	0.96	0.92	4.41
Bone area measures			
Forearm	0.82	0.66	0.16
FN	0.87	0.75	0.35
LS	0.88	0.76	4.64

BMD = bone mineral density; BMC = bone mineral content; FN = femoral neck; LS = lumbar spine

The Hologic BMD, BMC and BA data were further converted to the Lunar data using the following equations: forearm BMD Lunar = -0,085263 + 1,356535*Hologic; LS BMD Lunar = 0,030762 + 1,161805*Hologic; FN BMD Lunar = 0,084782 + 1,116509*Hologic; forearm BMC Lunar = 0,148564 + 1,117715*Hologic; LS BMC Lunar = 7,143123 + 0,923483*Hologic; FN BMC Lunar = 0,079107+ 1,106219*Hologic; BA forearm Lunar = 0,784022 + 0,683982*Hologic; BA LS Lunar = 6,843735 + 0,765959*Hologic; BA FN Lunar = -0,467152 + 1,023246*Hologic.

### Statistical analyses

Projected power to detect differences between dance students and controls were performed based on prior studies with similar cohorts and study design. Power calculations were conducted based on a sample of female dance students (n = 33, 16.2+2.0yr) and controls (n = 90, 16.6+1.0yr) with BMD at the femoral neck as the main outcome. Assuming a detectable difference of 0.4 standard deviation and 90% power, calculations indicated that a sample of 50 volunteers was required for the present cross-sectional study (25 dance students and 25 controls).

Independent t-tests were used to compare descriptive characteristics and crude values of bone measurements between dance students and controls. Bone parameters were compared after adjustment for Tanner stage, height, body mass and calcium intake using a three-factor analysis of covariance (ANCOVA). All residuals were tested for normal distribution using the Kolmogorov-Smirnov test. As the normality assumptions were not violated in the majority of sites, multiple regression analysis was performed to test for the association between bone measurements (dependent variable) with several independent variables known as predictors from literature (maturation, body weight, height, energy and nutrition intakes); each variable was additionally inserted in the same model as a stepwise manner–one model for each anatomical site. For all analyses the SPSS—version 20.0 (IBM SPSS, Chicago, IL) was used while statistical significance was set at *p<*0.05.

## Results

Dance students had significantly lower body weight and height than controls (*p*<0.001 and *p* = 0.001, respectively; Table 2). By the time of the assessment, two dance students and 15 controls had reached menarche (one dancer had oligomenorrhea, whereas the other dancer and all 15 controls had regular menses). A significantly higher number of dance students were at Tanner sexual pubertal development I (67.6%), while controls were at stage IV (40.0%), *p*<0.001. There was no significant difference in terms of calorie intake between groups, but daily calcium intake was significant greater in dancers (*p* = 0.03).

**Table 2:** Participant characteristics.

	Dance Students		Control Students
	(N = 34)		(N = 30)
Age (years) ^(1)^	10.9 ± 0.7	11.1 ± 0.5	
Height (cm) ^(1)^	143.8 ± 6.8**	150.4 ± 9.7	
Weight (Kg) ^(1)^	33.0 ± 5.8***	48.2 ± 9.9	
Dance training before vocational dance school (h/week) ^(1)^	1.8±0.7	—	
Age at menarche ^(2)^	10.5 ± 0.7	10.8 ± 0.9	
Amenorrhea ^(2)^	0.0	0.0	
Oligomenorrhea ^(2)^	0.34	0.0	
Calcium intake (mg/day) ^(1)^	839.2 ± 498.1*	664.8 ± 331.7	
Energy intake (Kcal/day) ^(1)^	1863.8 ± 498.7	1763.0 ± 339.0	
Tanner stage 1 ^(2)^	67.6	6.7	
Tanner stage 2 ^(2)^	32.4	30.0	
Tanner stage 3 ^(2)^	0.0	23.3	
Tanner stage 4 ^(2)^	0.0	40.0	

^(1)^ Values are means + SD

^(2)^ Values are percentages

* p<0.05

** p<0.01

*** p<0.001

Dance students displayed significantly lower (*p*<0.001) crude BMD and BMC values than controls at all measured sites (Table 3). Significantly lower BMAD values by 43.6% were also found at the FN in dancers compared to controls (*p*<0.001; Table 3), and 17.6% lower at the LS (*p*<0.001; Table 3). There were no significant differences in crude BMAD between the two groups at the forearm.

**Table 3:** Unadjusted bone parameters.

	Dance Students	Control Students	Relative Difference
	(N = 34)	(N = 30)	(%)
Forearm measures			
BMC (g)	1.22 ± 0.22	1.69 ± 0.29***	38.1
BMD (g/cm^2^)	0.54 ± 0.07	0.69 ± 0.07***	28.2
BMAD (g/cm^3^)	0.24 ± 0.32	0.29 ± 0.34	18.6
FN measures			
BMC (g)	2.95 ± 0.69	3.67 ± 0.71***	24.5
BMD (g/cm^2^)	0.81 ± 0.14	1.02 ± 0.09***	25.0
BMAD	0.19 ± 0.04	0.27 ± 0.04***	43.6
LS measures			
BMC (g)	29.07 ± 8.87	40.23 ± 11.38***	38.4
BMD (g/cm^2^)	0.76 ± 0.14	0.98 ± 0.14***	28.3
BMAD (g/cm^3^)	0.13 ± 0.04	0.15 ± 0.02***	17.6

Values are means + SD

* p<0.05

** p<0.01

*** p<0.001

BMC = bone mineral content; BMD = bone mineral density; BMAD = bone mineral apparent density; FN = femoral neck; LS = lumbar spine

Regression analyses revealed that when bone parameters were adjusted for body weight, height, pubertal development (Tanner test) and calcium intake, dance students continued to display significantly lower BMD at the forearm (*p* = 0.02) and FN (*p* = 0.04) (Table 3). For the same adjustments, BMAD values at the FN were also significantly lower in dancers than controls (*p*<0.001) (Table 4). The other remaining bone mass parameters were not significantly different between dancers and controls after the adjustment (Table 4).

**Table 4:** Adjusted bone parameters for Tanner stage, height, body weight and calcium intake.

	Dance Students	IC 95%	Control Students	IC 95%	Relative Difference
	(N = 34)		(N = 30)		(%)
Forearm measures					
BMC (g)	1.37 ± 0.06	1.25–1.49	1.56 ± 0.07	1.43–1.69	13.9
BMD (g/cm^2^)	0.59 ± 0.02	0.56–0.62	0.66 ± 0.02**	0.62–0.69	11.9
BMAD (g/cm^3^)	0.26 ± 0.39	0.13–0.39	0.27 ± 0.07	0.13–0.41	3.8
FN measures					
BMC (g)	3.38 ± 0.12	3.12–3.64	3.28 ± 0.14	2.98–3.57	3.0
BMD (g/cm^2^)	0.87 ± 0.03	0.82–0.92	0.96 ± 0.03*	0.92–1.04	10.3
BMAD	0.19 ± 0.07	0.17–0.21	0.27 ± 0.01***	0.25–0.29	42.1
LS measures					
BMC (g)	36.14 ± 2.04	32.05–40.22	32.47 ± 2.33	27.79–37.14	12.8
BMD (g/cm^2^)	0.87 ± 0.03	0.82–0.93	0.86 ± 0.03	0.80–0.93	6.1
BMAD (g/cm^3^)	0.14 ± 0.01	0.12–0.16	0.15 ± 0.01	0.13–0.17	7.1

Values are means + SD

* p<0.05

** p<0.01

*** p<0.001

BMC = bone mineral content; BMD = bone mineral density; BMAD = bone mineral apparent density; FN = femoral neck; LS = lumbar spine

Multiple regression analysis showed that body weight and height were significantly associated with BMC at the FN (R^2^ = 0.607; *p* = 0.03 and *p* = 0.01, respectively), whist Tanner stage was significantly associated with BMD at the LS (R^2^ = 0.654; *p* = 0.002). Height was also a significant predictor of BMAD at the FN (R^2^ = 0.637; *p* = 0.02), and Tanner stage a significant predictor of BMC at the LS (R^2^ = 0.600; *p* = 0.02) and BMD at the forearm (R^2^ = 0.710; *p* = 0.03). Calcium intake was significantly associated with BMAD measurements at the forearm (R^2^ = 0.100; *p* = 0.03).

## Discussion

To our knowledge, this is the first study which examined levels of areal and volumetric measures of bone mass in pre-pubertal female vocational dance students prior to undergoing any serious professional training. Our data revealed that first year vocational dancers demonstrated significantly lower adjusted BMD at the forearm, and significantly lower adjusted BMAD and BMD at the FN compared to aged- and sex-matched controls. It could be argued, therefore, that by the time our volunteers were selected to receive professional dance training they already demonstrated inferior bone mass measurements than controls.

It is difficult to compare the present findings with available data as the majority of the latter have examined professional dancers, non-elite adolescents or advanced vocational students [32–35]. This means that, unlike our participants, those involved in the aforementioned studies have already been exposed to the effects of dance training on the skeleton (they have been exercising for longer than two years). Also, previous studies on vocational dance students have reported mean ages of 16.7±0.8yr [14], 17.0±0.2yr [13, 36], 21.5±3.7yr [37] and 20.7±1.8yr [38], which are significantly higher than our cohort (10.9±0.7yr), while only one of these studies had estimated volumetric densities [14]. This estimation may be particular relevant as it reduces the effect of bone size on areal density [25]. It is therefore important to calculate volumetric densities when interpreting paediatric densitometry in order to avoid overestimating bone mass values in tall children and underestimate it in short children [25, 39]. Nevertheless, it should be highlighted that the studies on vocational dance students suggest that vocational dance training environment can lead to low body weight values, menstrual disturbances, diet restriction and, consequently, low bone weight phenotypes [13, 14, 37]. Indeed, participants involved in aesthetic activities, like elite dancing, have been identified as potentially at-risk to develop the so-called female athlete triad [40, 41]. However, our results indicate that prior to vocational training our dance students already demonstrated lower bone mass compared to controls, which might signify the presence of a dance-audition-selection bias.

It should be also highlighted that our vocational dancers were involved in extra exercise at young ages prior to their selection for vocational dance training. This extra exercise comprised approximately 1.8 hours per week of recreational dancing. It has been recently suggested that, during pre-puberty, early-puberty and puberty, the effects of exercise on bone mass in girls is minimal [42]. Also, although the exact frequency and duration of exercise that significantly affects bone metabolism warrants further investigation, the current recommendations for enhancing bone health in children is two or more times per day of 10–20 min of weight-bearing activities for at least 3 days per week [43]. Therefore, it seems unlikely that these 1.8 hours of exercise per week would have any effects on bone development.

Multiple regression analysis revealed that maturation and body type characteristics are likely to associate with bone mass parameters. Actually, the majority of our dance students were at Tanner sexual stage I, whereas controls were at stage IV. Our dancers were also significantly shorter and lighter than controls and only two had reached menarche, against 15 controls of the same age. Moreover, dancers had significant greater calcium intakes and total energy intake (despite not significant) than controls. Therefore, our results may suggest that children with a predisposition for low body weight and delayed maturation are selected for professional dance training. Indeed, genetic factors seem to account for the majority of the bone mass phenotypes [44]. It was showed that girls who experience later menarche also have low values of BMD during pre-puberty [45], suggesting that genetics is a determinant factor. However, this issue needs to be further investigated in dancers. Longitudinal research protocols should also be used to establish whether such bone mass values would have any bearing to peak bone mass, which is an important factor for prevention of bone fracture and osteoporosis [46, 47].

One of the strengths of the current study is the representativeness of its sample given the relatively large number of vocational dance students who volunteered. This issue answers one of the main criticisms regarding dancers´ bone health which is related to the relatively small studied cohorts [18]. Another strength of this study is the young age of our participants; first year vocational dance students have never been studied before in relation to bone health prior to professional dance training. A further strength might be the confounding variables used to analyse bone mass results, which have not been frequently considered in the past [18]. This is also the case with the effect of bone size on DXA measurements; therefore, bone mass data presented in terms of BMC, BMD and BMAD is another strength of the present study.

It is reasonable to assume that the present results may have been influenced by methodological limitations. For example, due to the study’s observational nature, causality and changes through time cannot be established. The use of Tanner staging is an also limitation; the assessment of skeletal maturation through X-ray at the hand-wrist bones would have been more accurate. However, ethical issues has prevented us of doing so given that such a X-ray would have been an additional radiation exposure to that already coming from DXA scans. Given the well-known role of endocrine mechanisms in bone mass acquisition during the growing years, the lack of hormonal data is a further limitation of the present study. Self-reported nutrition data is also acknowledged as a shortcoming. Finally, the use of two different DXA scans to assess participants and the need to adjust the data for potential bias is a limitation. Nevertheless, this approach has been previously used and it is deemed acceptable for studies of this kind [48, 49].

## Conclusions

Prior to commencing full professional dance training, first year female vocational dance students demonstrate low bone mass parameters compared to aged- and sex-matched controls. Therefore, the low BMD values reported in professional dancers might have their genesis during the growing years. Further longitudinal research is required to ascertain how bone mass parameters change with time throughout professional dance training.
